# Scaling neighbor joining to one million taxa with dynamic and heuristic neighbor joining

**DOI:** 10.1093/bioinformatics/btac774

**Published:** 2022-12-01

**Authors:** Philip T L C Clausen

**Affiliations:** Research Group for Genomic Epidemiology, National Food Institute, Technical University of Denmark, 2800 Kgs. Lyngby, Denmark

## Abstract

**Motivation:**

The neighbor-joining (NJ) algorithm is a widely used method to perform iterative clustering and forms the basis for phylogenetic reconstruction in several bioinformatic pipelines. Although NJ is considered to be a computationally efficient algorithm, it does not scale well for datasets exceeding several thousand taxa (>100 000). Optimizations to the canonical NJ algorithm have been proposed; these optimizations are, however, achieved through approximations or extensive memory usage, which is not feasible for large datasets.

**Results:**

In this article, two new algorithms, dynamic neighbor joining (DNJ) and heuristic neighbor joining (HNJ), are presented, which optimize the canonical NJ method to scale to millions of taxa without increasing the memory requirements. Both DNJ and HNJ outperform the current gold standard methods to construct NJ trees, while DNJ is guaranteed to produce exact NJ trees.

**Availability and implementation:**

https://bitbucket.org/genomicepidemiology/ccphylo.git

**Supplementary information:**

Supplementary data are available at *Bioinformatics* online.

## 1 Introduction

Hierarchical clustering methods are widely used across several scientific fields and form an essential part of unsupervised machine learning (Lord *et al.*, 2015; Nagpal *et al.*, 2013; Shirkhorshidi *et al.*, 2014). A key advantage of hierarchical clustering, compared with other clustering methods, is the ability to perform clustering without assumptions on the number of clusters (such as with *k*-means clustering; Forgy, 1965) nor does it need a minimum distance for data points to cluster together [such as Hobohm (Hobohm *et al.*, 1992) and DBSCAN (Ester *et al.*, 1996)]. In addition, a hierarchical clustering opens the possibility to apply algorithms such as HDBSCAN (Campello *et al.*, 2013), allowing the test of several density thresholds in a computational efficient manner.

In the field of bioinformatics, neighbor joining (NJ) (Saitou and Nei, 1987) is often used to infer approximate phylogenies of large datasets and is used as the base tree for several maximum-likelihood phylogenetic methods (Minh *et al.*, 2020; Price *et al.*, 2010). While NJ is considered to be a computationally efficient algorithm, it has problems scaling to the size of datasets seen today and is not feasible for datasets exceeding several thousand taxa (>100 000). Distance matrices exceeding several thousand entries can be constructed and updated efficiently today using software such as MASH, Minimap2, MINTyper, KMA and EverGreen (Clausen *et al.*, 2018; Hallgren *et al.*, 2021; Li, 2018; Ondov *et al.*, 2016; Szarvas *et al.*, 2020), which have been proven through the global and national surveillance of bacterial pathogens and SARS-CoV-2 (du Plessis *et al.*, 2021; Szarvas *et al.*, 2020; Timme *et al.*, 2018; Wu *et al.*, 2020).

Over the years, several optimizations have been proposed to speed up the canonical NJ algorithm, such as relaxed neighbor joining (RNJ) (Evans *et al.*, 2006), fast neighbor joining (FNJ) (Elias and Lagergren, 2009), RapidNJ (Simonsen *et al.*, 2008) and NINJA (Wheeler, 2009). RNJ relaxes the join criterion, which allows for early stopping, although reducing the practical runtime to approximately *n*^2^log(*n*); the worst-case runtime remains *O*(*n*^3^) (Evans *et al.*, 2006). In difference to RNJ, FNJ guarantees a quadric runtime by identifying the optimal join criterion of each row in the distance matrix prior to clustering, which is then kept in the remaining iterations (Elias and Lagergren, 2009). RapidNJ and NINJA guarantee exact NJ trees, unlike RNJ and FNJ that only guarantee this for additive distance matrices. Through sorting of the distance matrix, RapidNJ and NINJA are able to apply a lossless early stopping criterion by keeping track of the relations between the sorted and unsorted distance matrix. For most datasets, RapidNJ and NINJA achieve a practical runtime of *n*^2^log(*n*), although worst case remains at *O*(*n*^3^). Due to maintaining both sorted and unsorted data access capability, RapidNJ and NINJA require an additional two matrices to be stored, which increases the memory consumption three to six times compared with RNJ and FNJ, depending on the implementation.

In this article, two new algorithms, dynamic neighbor joining (DNJ) and heuristic neighbor joining (HNJ), are presented that outperform the gold standard methods for exact and approximate NJ on both speed and memory requirements. In addition, it is proven that DNJ is guaranteed to produce exact NJ trees.

## 2 Materials and methods

### 2.1 Data

Distance matrices were collected and constructed from various sources with varying sizes from a few thousand samples up to one million samples (see Table 1 and Supplementary Table S1).

**Table 1.** Overview of test data, see Supplementary Table S1 for an extended version of the table including links to the data

Dataset	Sample type	Distance measure	Number of samples
ResFinder	Antimicrobial resistance genes	Jaccard distance	3160
KmerFinder	Complete bacterial genomes	Jaccard distance	23 331
Krummholz	SARS-CoV-2	Hamming distance	129 260
COG-417K	SARS-CoV-2	SNP distance	417 947
COG-664K	SARS-CoV-2	SNP distance	664 632
Chevrier	Mass cytometry	Cosine distance	1 000 000

The Jaccard distance was calculated on indexed *k-*mers for the ResFinder (Clausen *et al.*, 2018; Zankari *et al.*, 2012) and KmerFinder (Clausen *et al.*, 2018; Sayers *et al.*, 2019) databases using KMA (Clausen *et al.*, 2018), including a total of 3160 antimicrobial resistance genes and 23 331 complete bacterial genomes, respectively. A distance matrix containing the Hamming distance between 129 260 SARS-CoV-2 samples across the world was included, which was constructed with the Krummholz pipeline, a modified version of EverGreen (Clausen *et al.*, 2018; Szarvas *et al.*, 2020) (described at: https://covid-phylogeny.genomicepidemiology.org/about). Two additional datasets of SARS-CoV-2 were included from the COG-UK surveillance program, containing 417 947 and 664 632 samples in Newick format constructed with the Phylopipe pipeline (described at: https://github.com/cov-ert/phylopipe), which were converted to strictly additive distance matrices. Mass cytometry data of 32 proteins were included from Chevrier et al. (2017), including a total of 1 814 325 cells (Chevrier *et al.*, 2017). These were downsampled by taking the first one million non-null vector readings, and a distance matrix was constructed using the cosine distance measure.

All distance matrices were formatted as relaxed lower triangular PHYLIP matrices.

### 2.2 Dynamic neighbor joining

The NJ method (Saitou and Nei, 1987) is an iterative clustering method that iteratively joins nodes by minimizing:

(1) $$Q_{i,j}=\;\left(n-2\right)D_{i,j}-S_{i}-S_{j},\;S_{i}=\sum_{k\leq n}D_{i,k}\;$$

where *n* is the remaining number of nodes left and *D_i_*_,__*j*_ is the distance between nodes *i* and *j*. The distance between a newly formed node and all other nodes is then computed as:

(2) $$D_{i,z}=\frac{D_{x,i}+D_{i,y}-D_{x,y}}{2}\;$$

where *z* is the newly formed node, formed by joining nodes *x* and *y*. Applying Equations (1) and (2) iteratively will gradually increase the join criterion presented in (1), under the assumption that the newly formed node will not lead to a better join than the previous best join for each row (see Lemma 1).

Lemma 1 Given a distance matrix *D* where node pair (*x*, *y*) minimizes (1) for all possible node pairs and node pair (*i*, *j*) which minimizes row *i*, thus, an alternative node pair (*i*, *k*) will not exist giving smaller join criterion following (1) after joining the node pair (*x*, *y*), when excluding the newly formed node (*z*) from joining the node pair (*x*, *y*) for all *k*. In other words, the join criterion through (1) will be gradually weakened through the iterative joins performed. This observation will thus satisfy:

$$\left(n-2\right)D_{i,j}-S_{i}-S_{j}\leq {\left(\hat{n}-2\right)D}_{i,k}-{\hat{S}}_{i}-{\hat{S}}_{k}$$

where *n*, *D* and *S* are defined as in (1), while $\hat{n}$ and $\hat{S}$ denotes *n* and *S* after joining node *x* and *y*.

**Proof:**

The above inequality can be rewritten as:

$$\left(n-2\right)D_{i,j}-S_{i}-S_{j}\leq {\left(\left(n-1\right)-2\right)D}_{i,k}-\left(S_{i}-D_{x,i}-D_{i,y}+D_{i,z}\right)\;-(S_{k}-D_{x,k}-D_{k,y}+D_{k,z})$$

$$⟺\left(n-2\right)D_{i,j}-S_{i}-S_{j}\leq {\left(\left(n-1\right)-2\right)D}_{i,k}\;-{(S}_{i}-D_{x,i}-D_{i,y}+\frac{D_{x,i}+D_{i,y}-D_{x,y}}{2})-{(S}_{k}-D_{x,k}-D_{k,y}+\frac{D_{x,k}+D_{k,y}-D_{x,y}}{2})$$

$$⟺\left(n-2\right)D_{i,j}-S_{i}-S_{j}\leq \left(n-2\right)D_{i,k}-S_{i}-S_{k}\;+D_{x,y}-D_{i,k}+\frac{D_{x,i}+D_{i,y}}{2}+\frac{D_{x,k}+D_{k,y}}{2}$$

Knowing that $\left(n-2\right)D_{i,j}-S_{i}-S_{j}\leq {\left(n-2\right)D}_{i,k}-S_{i}-S_{k}$, the equation can be rewritten as below, which is satisfied through the triangle inequality, and non-negative distances.

$$0\leq {2D}_{x,y}+\left(D_{i,x}+D_{x,k}-D_{i,k}\right)+{(D}_{i,y}+D_{y,k}-D_{i,k})$$

Using this observation, Equation (1) can be rewritten to the dynamic equation below, making the foundation of DNJ.

(3) $$Q_{i}=\;\left\{\begin{array}{ll} \underset{k\;<\;i}{\min}\left\{\left(n-2\right)*D_{i,k}-S_{i}-S_{k}\right\} & Q_{i}<M_{i+1}\\ Q_{i}\; & e\mathrm{lse} \end{array}\right.\;$$

(4) Mi=minQiMi+1 

where *Q* is a vector containing the optimal join criteria of each row in the triangular distance matrix *D*, *M* is used to keep track of the global minimum join criteria, where *M_n_*_+1_ is set to ∞. *Q* can then be updated through the equation below, to satisfy the assumption made in Lemma 1.

(5) Qi=minQin-2Di,z-Si-Sz, z<i

where *z* is the newly formed node, *Q_z_*, *S* and *D* are all updated in linear time prior to applying Equation (5). The minimum join criterion is then identified as the argument minimizing *Q* after searching a row through Equation (3).

A slight optimization may be applied to Equation (4) by initializing *M_n_*_+1_ to *Q_z_*, as the newly formed node is likely to minimize Equation (3).

Intuitively, this implementation requires fewer rows to be examined in *D*, as $Q_{i}\ll Q_{k}$ for most k≠i where *Q_i_* contains the optimal join criterion. Effectively, this reduces the time complexity of minimizing *Q* from *O*(*n*^2^) to *O*(*dn*), where *d* is the number of rows in *D* that needs to be examined. Thus, giving a total time complexity of the entire algorithm of *O*(*dn*^2^) while guaranteeing exact NJ trees, with space complexity *O*(*n*^2^) as only one triangular matrix needs to be stored.

As for the NJ algorithm, the update of *Q* [Equation (3)] can be parallelized, however, with reduced efficiency, as only a limited number of rows are visited in each iteration, together with the possibility of examining unnecessary rows.

### 2.3 Heuristic neighbor joining

Asymptotically, DNJ reaches a runtime of *O*(*n*^3^) when updates to *D* [through equation (2)] cause frequent updates in *Q* [through Equation (3)]. This worst-case time complexity can be reduced to *O*(*n*^2^) with an approximating search heuristic, giving rise to HNJ. As for DNJ, HNJ initializes *Q* with the minima of each row in *D* and updates *D* with Equation (2). Different to DNJ, HNJ updates *Q* with the equation below and joins nodes by minimizing *Q* without applying Equation (3).

(6) Qi=minn-2Di,j-Si-Sjn-2Di,z-Si-Sz, z<i ∧ j<i 

where *j* is the node minimizing Equation (1) in the initialization step, and *z* is the newly formed node.

Applying Equation (6) over (3) and (5) will produce exact NJ trees only in the case where a join between two nodes will not lead to an alternative minimum through Equation (1) after updating *D*. In other words, there might exist a row for which the following applies after updating *D*.

$$\left(n-2\right)D_{i,k}-S_{i}-S_{k}<\left(n-2\right)D_{i,j}-S_{i}-S_{j}$$

where *j* is the node minimizing Equation (1) in the initial round of NJ and *k* is an alternative node different from the newly formed node. For strictly additive trees, this will never be satisfied.

Effectively, this reduces the time complexity of HNJ to *O*(*n*^2^), while the space complexity remains at *O*(*n*^2^) as for DNJ.

## 3 Results and discussion

**Fig. 1.**
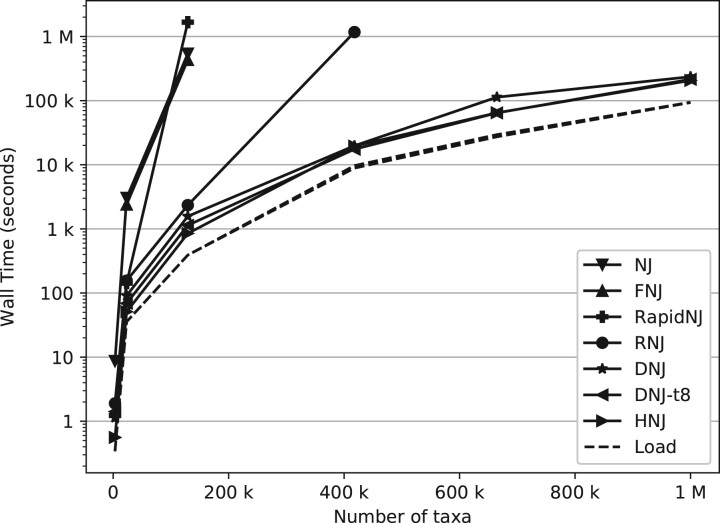
Run time comparison using real data from Table 1 on an Intel^®^ Xeon^®^ Gold 6230 CPU with 512 GB memory, between NJ, FNJ, RapidNJ, RNJ, HNJ and DNJ using one and eight threads (DNJ and DNJ-t8, respectively). Note that the input data were gzipped for the dataset containing 1 M taxa. The ‘Load’ illustrates the time interval used to load the distance matrix, measured through DNJ and HNJ

**Fig. 2.**
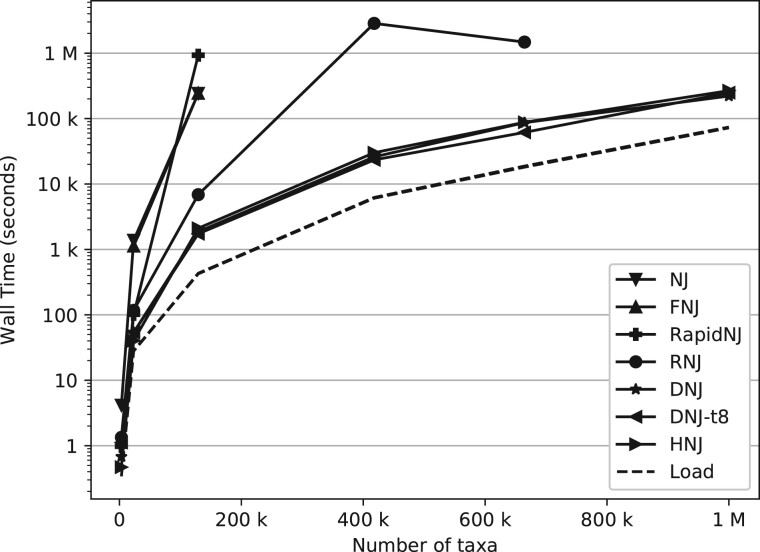
Run time comparison using real data from Table 1 on an AMD EPYC 7352 24-Core Processor with 2 TB memory, between NJ, FNJ, RapidNJ, RNJ, HNJ and DNJ using one and eight threads (DNJ and DNJ-t8, respectively). Note that the input data were gzipped for the dataset containing 1 M taxa. The ‘Load’ illustrates the time interval used to load the distance matrix, measured through DNJ and HNJ

DNJ and HNJ were added to the CCPhylo software suite (v0.6.0) and compared with FNJ through fastphylo (v1.0.1) (Khan *et al.*, 2013), RapidNJ (v2.3.2) (Simonsen *et al.*, 2008), NINJA (v1.2.2) (Wheeler, 2009), NJ and RNJ through Clearcut (v1.0.9) (Sheneman *et al.*, 2006) using the data in Table 1. All methods were tested on two separate machines, one with an Intel^®^ Xeon^®^ Gold 6230 CPU and 512 GB memory and one with an AMD EPYC 7352 24-core Processor and 2 TB memory, and timed using GNU time (see Figs. 1 and 2 and Supplementary Tables S2 and S3). The DNJ and HNJ executions were set to use float precision to match that of the other methods, 2- and 1-byte precision was used for HNJ and DNJ on the COG-664K and Chevrier datasets, respectively, due to memory limitations on the Intel machine (512 GB available). FNJ exceeded the memory limits for the datasets larger than Krummholz. The NJ method was excluded for these datasets as well, due to the expected runtime. RapidNJ was forcefully terminated on the COG-417K due to an excessive runtime (>3 months). The Chevrier distance matrix was gzipped due to the limitations of the disk, with a max file size of 4 TB. The remaining options were left as default, except for DNJ where the threading performance was tested with eight threads (see Supplementary Tables S2–S4 for more details). The distance matrices were transformed to full-formatted distance matrices for testing with FNJ, RapidNJ and NINJA, as these implementations were limited to full-PHYLIP matrices only.

HNJ outperformed RNJ by a factor of at least three and more than an order of magnitude for the COG-417K and COG-664K datasets. The difference between HNJ and DNJ was more subtle and varied with a factor of zero to one, where this difference was limited when adding threads to the DNJ algorithm. The algorithms implemented through Clearcut (NJ and RNJ) and CCPhylo (DNJ and HNJ) had a similar memory consumption, varying by a few MBs only. FNJ and RapidNJ had the highest memory usage, being approximately eight and four times that of the remaining methods, respectively (see Supplementary Tables S2 and S3).

The bottleneck of DNJ and HNJ was identified as the loading of the distance matrix for all of the tested datasets, which accounted for about half of the execution time on most datasets (see Figs. 1 and 2 and Supplementary Tables S2 and S3). This indicates a constant value of *d* for the *O*(*dn*^2^) runtime of DNJ on the tested datasets, reducing the effective runtime of DNJ to quadric time (*O*(*n*^2^)). It should be noted that in case of star shaped trees with uniform branch lengths, the effectiveness of DNJ would be limited with *d ≈ n*. This worst-case performance, with *d ≈ n*, can however be disregarded as a practical concern, as this kind of data would not compose a hierarchical nor iterative clustering problem.

FNJ crashed with a segmentation fault on the ResFinder dataset. NINJA produced invalid trees for all datasets, while returning exit code zero with no warnings nor errors.

As seen from Figures 1 and 2 and Supplementary Table S2 and S3, the implementation itself has a great impact on the runtime of the various algorithms. As FNJ and HNJ both guarantee *O*(*n*^2^) runtime, these were expected to be the fastest, however, the measured runtimes of FNJ were closer to that of the canonical runtime than any of the other methods. Similarly, it is seen that RapidNJ and RNJ have a considerably longer runtime than expected when analyzing the Krummholz and COG-417K datasets, respectively. A closer inspection of the implementation of RapidNJ revealed that the matrices were not shrunken gradually, which caused an extensive amount of page faults when analyzing larger datasets. The search heuristics performed with RNJ are challenged by imbalanced trees (Evans *et al.*, 2006), as is the case for SARS-CoV-2 (Duarte *et al.*, 2022).

The size of the approximations made by HNJ, RNJ and FNJ was evaluated using the Robinson–Foulds (RF) distance between the exact NJ trees produced by DNJ and the remaining tested methods (see Supplementary Tables S2 and S3), using IQ-Tree (v2.0.3) (Minh *et al.*, 2020). RNJ was closer to the exact NJ tree than HNJ on the ResFinder dataset with a greater margin than that indicated by comparing the exact NJ trees. RNJ, FNJ and HNJ were as distant to the exact NJ tree on the KmerFinder dataset as the individual exact methods were. The greatest distances between RNJ, FNJ and HNJ to the exact tree were observed on the Krummholz dataset with a normalized RF of [0.220; 0.253]. IQ-Tree became unstable with the data size encountered on the COG-417K dataset and was not applied to the COG-664K and Chevrier datasets. The metric would have been of limited value for the COG-417K and COG-664K datasets, as these were strictly additive datasets resulting in exact NJ trees for all tested methods.

Reducing the distance precision to one byte on the Chevrier dataset will naturally limit the performance of the algorithm, although the effect on the tree topology will be limited as both *S* and *Q* are stored using double precision. This allows a total of (2*n* − 2)256 possible join values through Equation (1). The internal branch lengths will however be largely affected, as only 256 distance values are available using byte precision.

With the development of DNJ and HNJ, hierarchical clustering is no longer limited by the computation time, but by the sheer size of the distance matrix traditionally used. Further development on hierarchical clustering algorithms will therefore need to utilize the data directly, for example, by inferring and calculating the distances only to affected nodes while updating the tree, similar to that proposed in SLINK and PartTree (Katoh and Toh, 2007; Sibson, 1973).

The improvements to the canonical NJ presented through DNJ and HNJ have scaled the construction of NJ trees to millions, making the size of distance matrix compared with the available memory and disk size the new limiting factor.

## Supplementary Material

btac774_Supplementary_Data

## Data Availability

DNJ and HNJ have been included in the CCPhylo software suite (as ‘CCPhylo tree’), as well as UPGMA and a hierarchical Furthest First clustering optimized with the same algorithm as for DNJ. Likewise, Closest First is a part of CCPhylo that has a lossless time complexity of *O*(*n*^2^). The memory requirement remains at *O*(*n*^2^) and is dominated by the lower triangular distance matrix, which can be stored with double, float, 2- or 1-byte precision, set through runtime options, as well as the possibility to store the matrix on disk (CCPhylo: https://bitbucket.org/genomicepidemiology/ccphylo.git; operating system(s): Unix-based systems; programming language: C; other requirements: zlib development files; and license: Apache v2.0).
