# Supplementary material for: Scaling neighbor joining to one million taxa with dynamic and heuristic neighbor joining
Source: Bioinformatics. 2022 Dec 1;39(1):btac774. doi: 10.1093/bioinformatics/btac774 (PMC9805563; doi:10.1093/bioinformatics/btac774)
Supplement: btac774_Supplementary_Data [file btac774_supplementary_data.zip › btac774_Supplementary_Data/S1.pdf]

Dataset	Sample type	Distance measure	Number of samples	Source
**ResFinder**	Antimicrobial resistance genes	Jaccard distance	3160	https://sciencedata.dk/shared/d1e5a0e1916777f87c9d3cb765647a3e?download (https://bitbucket.org/genomicepidemiology/resfinder_db.git Commit: eec8752)
**KmerFinder**	Whole bacterial genomes	Jaccard distance	23331	https://sciencedata.dk/shared/8da6465076fa9e75197a4ccf1b2b7d07?download
**Krummholz**	SARS-CoV-2	Hamming distance	129260	https://sciencedata.dk/shared/62980321c6a4096dc0bf5e11026402ac?download
**COG-417K**	SARS-CoV-2	SNP distance	417947	https://sciencedata.dk/shared/54290ad58cb3b2524f0c01470a53a1b1?download
**COG-664K**	SARS-CoV-2	SNP distance	664632	https://sciencedata.dk/shared/258a905ba7dbc7f3481810903c94aebb?download
**Chevrier**	Mass Cytometry	Cosine distance	1000000	https://premium.cytobank.org/cytobank/projects/875

Table S1; Overview of test data.
