# Supplementary material for: Scaling neighbor joining to one million taxa with dynamic and heuristic neighbor joining
Source: Bioinformatics. 2022 Dec 1;39(1):btac774. doi: 10.1093/bioinformatics/btac774 (PMC9805563; doi:10.1093/bioinformatics/btac774)
Supplement: btac774_Supplementary_Data [file btac774_supplementary_data.zip › btac774_Supplementary_Data/S2.pdf]

Method	Command	RF	Version	Peak Memory	CPU Time (h:mm:ss)	Wall Time (h:mm:ss or m:ss)
**ResFinder (n = 3160, lt ∊ [0:00:0.34 ; 0:00:0.35])**						
NJ	clearcut --neighbor	420 (0.067)	1.0.9	**20.27 MB**	0:00:08.67	0:08.69
RapidNJ	Rapidnj	498 (0.079)	2.3.2	93.98 MB	0:00:01.21	0:01.23
NINJA	Ninja –in_type d	n/a	0.95	268.92 MB	0:00:22.36	0:22.40
FNJ	fnj -I phylip -O newick	n/a	1.0.1	155.62 MB	0:00:04.78	0:04.82
RNJ	clearcut	774 (0.123)	1.0.9	20.32 MB	0:00:01.89	0:01.90
DNJ	ccphylo tree -p	0 (0)	0.6.0	21.79 MB	0:00:01.13	0:01.14
DNJ^{t8}	ccphylo tree -pt 8	0 (0)	0.6.0	21.93 MB	0:00:02.51	0:01.42
HNJ	ccphylo tree -pm hnj	1304 (0.207)	0.6.0	21.86 MB	**0:00:00.54**	**0:00.56**
**KmerFinder (n = 23331, lt ∊ [0:00:35.63 ; 0:00:35.70])**						
NJ	clearcut --neighbor	2358 (0.051)	1.0.9	**1.02 GB**	0:50:37.61	50:37.79
RapidNJ	Rapidnj	4578 (0.098)	2.3.2	4.23 GB	0:02:22.56	2:22.85
NINJA	Ninja –in_type d	n/a	0.95	19.39 GB	2:52:59.49	2:53:05
FNJ	fnj -I phylip -O newick	5340 (0.114)	1.0.1	8.16 GB	0:39:55.60	39:56.11
RNJ	clearcut	2728 (0.058)	1.0.9	**1.02 GB**	0:02:35.89	2:35.91
DNJ	ccphylo tree -p	0 (0)	0.6.0	**1.02 GB**	0:01:40.82	1:30.83
DNJ^{t8}	ccphylo tree -pt 8	0 (0)	0.6.0	**1.02 GB**	0:02:21.08	1:09.08
HNJ	ccphylo tree -pm hnj	4810 (0.103)	0.6.0	**1.02 GB**	**0:00:51.09**	**0:51.10**
**Krummholz (n = 129260, lt ∊ [0:06:30.17 ; 0:06:30.61])**						
NJ	clearcut --neighbor	21252 (0.082)	1.0.9	**31.14 GB**	149:41:49.57	149:42:06
RapidNJ	Rapidnj	25862 (0.100)	2.3.2	153.16 GB	464:26:29.54	464:27:00
NINJA	Ninja –in_type d	n/a	0.95	-	-	-
FNJ	fnj -I phylip -O newick	56830 (0.220)	1.0.1	249.16 GB	120:04:57.84	120:06:16
RNJ	clearcut	65492 (0.253)	1.0.9	**31.14 GB**	0:39:01.54	39:01.61
DNJ	ccphylo tree -p	0 (0)	0.6.0	31.15 GB	0:26:11.71	26:11.82
DNJ^{t8}	ccphylo tree -pt 8	0 (0)	0.6.0	31.15 GB	0:46:18.14	18:57.96
HNJ	ccphylo tree -pm hnj	62000 (0.240)	0.6.0	31.15 GB	**0:14:08.88**	**14:08.95**
**COG-417K (n = 417947, lt ∊ [2:27:41.79 ; 2:41:33.38])**						
RapidNJ	Rapidnj	n/a	2.3.2	-	-	-
NINJA	Ninja –in_type d	n/a	0.95	-	-	-
FNJ	fnj -I phylip -O newick	n/a	1.0.1	-	-	-

RNJ	clearcut	n/a	1.0.9	**325.43 GB**	282:05:12.82	282:08:17
DNJ	ccphylo tree -p	0 (0)	0.6.0	325.49 GB	**5:20:03.49**	5:22:27
DNJ^{t8}	ccphylo tree -pt 8	n/a	0.6.0	325.49 GB	9:53:42.27	**4:50:58**
HNJ	ccphylo tree -pm hnj	n/a	0.6.0	325.48 GB	5:21:37.23	5:25:37
**COG-664K (n = 664632, lt ∈ [7:34:41.58 ; 8:11:24.76])**						
RapidNJ	Rapidnj	n/a	2.3.2	-	-	-
NINJA	Ninja –in_type d	n/a	0.95	-	-	-
FNJ	fnj -I phylip -O newick	n/a	1.0.1	-	-	-
RNJ	clearcut	n/a	1.0.9	-	-	-
DNJ	ccphylo tree -s 65536	0 (0)	0.6.0	411.63 GB	31:14:57.58	31:24:10
DNJ^{t8}	ccphylo tree -s 65536 -t 8	n/a	0.6.0	411.63 GB	43:14:29.90	**17:50:36**
HNJ	ccphylo tree -s 65536 -m hnj	n/a	0.6.0	**411.62 GB**	**17:52:07.99**	17:57:15
**Chevrier (n = 1000000, lt ∈ [25:41:53.95 ; 26:08:39.73])**						
RapidNJ	rapidnj	n/a	2.3.2	-	-	-
NINJA	Ninja –in_type d	n/a	0.95	-	-	-
FNJ	fnj -I phylip -O newick	n/a	1.0.1	-	-	-
RNJ	clearcut	n/a	1.0.9	-	-	-
DNJ	ccphylo tree -b 255	0 (0)	0.6.0	465.90 GB	65:24:50.04	65:29:42
DNJ^{t8}	ccphylo tree -b 255 -t 8	0 (0)	0.6.0	465.90 GB	114:21:06.82	59:39:39
DNJ^{t32}	ccphylo tree -b 255 -t 32	0 (0)	0.6.0	465.90 GB	218:40:38.50	58:33:34
HNJ	ccphylo tree -b 255 -m hnj	n/a	0.6.0	**465.89 GB**	**57:08:29.82**	**57:14:13**

Table S2; Computational requirements of NJ, RapidNJ, NINJA, FNJ, RNJ, DNJ and HNJ, on an Intel® Xeon® Gold 6230 CPU with 512 GB memory. RF: Robinson-Foulds distance calculated with IQ-Tree and RAXML. n: number of taxa in dataset. lt: Time interval to load distance matrix (measured from ccphylo). t8: 8 threads, t32: 32 threads. Bold indicates lowest time or lowest peak memory for each dataset, measured with GNU time. -: Method terminated gracefully due to insufficient memory. Text in red indicates failed run or invalid output.
