# Supplementary material for: Scaling neighbor joining to one million taxa with dynamic and heuristic neighbor joining
Source: Bioinformatics. 2022 Dec 1;39(1):btac774. doi: 10.1093/bioinformatics/btac774 (PMC9805563; doi:10.1093/bioinformatics/btac774)
Supplement: btac774_Supplementary_Data [file btac774_supplementary_data.zip › btac774_Supplementary_Data/S3.pdf]

Method	Command	RF	Version	Peak Memory	CPU Time (h:mm:ss)	Wall Time (h:mm:ss or m:ss)
**ResFinder (n = 3160, lt ∈ [0:00:0.34 ; 0:00:0.34])**						
NJ	clearcut --neighbor	420 (0.067)	1.0.9	**20.43 MB**	0:00:04.14	0:04.15
RapidNJ	Rapidnj	498 (0.079)	2.3.2	96.16 MB	0:00:00.96	0:00.98
NINJA	Ninja –in_type d	n/a	0.95	263.77 MB	0:00:19.50	0:19.54
FNJ	fnj -I phylip -O newick	n/a	1.0.1	158.53 MB	0:00:02.89	0:03.07
RNJ	clearcut	779 (0.123)	1.0.9	21.47 MB	0:00:01.32	0:01.34
DNJ	ccphylo tree -p	0 (0)	0.6.0	23.24 MB	0:00:00.68	0:00.68
DNJ^{t8}	ccphylo tree -pt 8	0 (0)	0.6.0	23.74 MB	0:00:01.45	0:01.12
HNJ	ccphylo tree -pm hnj	1304 (0.207)	0.6.0	23.34 MB	**0:00:00.44**	**0:00.47**
**KmerFinder (n = 23331, lt ∈ [0:00:27.91 ; 0:00:28.23])**						
NJ	clearcut --neighbor	2358 (0.051)	1.0.9	**1.02 GB**	0:22:57.97	22:59.46
RapidNJ	Rapidnj	4578 (0.098)	2.3.2	4.24 GB	0:01:41.94	1:41.99
NINJA	Ninja –in_type d	n/a	0.95	19.37 GB	2:12:22.11	2:12:28
FNJ	fnj -I phylip -O newick	5340 (0.114)	1.0.1	8.16 GB	0:18:45.95	18:46.24
RNJ	clearcut	2852 (0.061)	1.0.9	**1.02 GB**	0:01:57.16	1:57.23
DNJ	ccphylo tree -p	0 (0)	0.6.0	**1.02 GB**	0:00:54.01	0:54.06
DNJ^{t8}	ccphylo tree -pt 8	0 (0)	0.6.0	**1.02 GB**	0:01:28.34	0:53.73
HNJ	ccphylo tree -pm hnj	4810 (0.103)	0.6.0	**1.02 GB**	**0:00:40.36**	**0:40.38**
**Krummholz (n = 129260, lt ∈ [0:07:03.68 ; 0:07:12.40])**						
NJ	clearcut --neighbor	21252 (0.082)	1.0.9	**31.14 GB**	67:12:01.33	67:17:47
RapidNJ	Rapidnj	25862 (0.100)	2.3.2	129.35 GB	258:03:32.82	258:18:22
NINJA	Ninja –in_type d	n/a	0.95	264.95 GB	136:33:02.27	136:36:03
FNJ	fnj -I phylip -O newick	56830 (0.220)	1.0.1	249.16 GB	67:40:59.16	67:42:28
RNJ	clearcut	65506 (0.253)	1.0.9	**31.14 GB**	1:54:30.34	1:54:35
DNJ	ccphylo tree -p	0 (0)	0.6.0	31.15 GB	**0:31:06.30**	31:07.52
DNJ^{t8}	ccphylo tree -pt 8	0 (0)	0.6.0	31.15 GB	0:48:26.41	**28:52.78**
HNJ	ccphylo tree -pm hnj	62000 (0.240)	0.6.0	31.15 GB	0:35:05.89	35:07.31
**COG-417K (n = 417947, lt ∈ [1:42:00.94 ; 1:43:47.85])**						
RapidNJ^{T}	Rapidnj	n/a	2.3.2	≥826.92 GB	>2544:47:41.98	>2547:11:14
NINJA	Ninja –in_type d	n/a	0.95	-	-	-
FNJ	fnj -I phylip -O newick	n/a	1.0.1	-	-	-

RNJ	clearcut	n/a	1.0.9	**325.44 GB**	790:39:49.67	792:00:15
DNJ	ccphylo tree -p	0 (0)	0.6.0	325.49 GB	**7:12:53.72**	7:13:11
DNJ^{t8}	ccphylo tree -pt 8	n/a	0.6.0	325.49 GB	11:33:20.81	**6:29:22**
HNJ	ccphylo tree -pm hnj	n/a	0.6.0	325.48 GB	8:21:31.46	8:21:52
**COG-664K (n = 664632, lt ∊ [5:01:40.24 ; 5:13:21.36])**						
RapidNJ	Rapidnj	n/a	2.3.2	-	-	-
NINJA	Ninja –in_type d	n/a	0.95	-	-	-
FNJ	fnj -I phylip -O newick	n/a	1.0.1	-	-	-
RNJ	clearcut	n/a	1.0.9	**822.91 GB**	409:56:37.03	410:16:08
DNJ	ccphylo tree -p	0 (0)	0.6.0	823.03 GB	**24:02:12.97**	24:03:47
DNJ^{t8}	ccphylo tree -pt 8	n/a	0.6.0	823.03 GB	28:37:47.23	**17:05:24**
HNJ	ccphylo tree -pm hnj	n/a	0.6.0	823.02 GB	24:18:41.19	24:20:54
**Chevrier (n = 1000000, lt ∊ [20:01:07.24 ; 20:37:08.85])**						
RapidNJ	Rapidnj	n/a	2.3.2	-	-	-
NINJA	Ninja –in_type d	n/a	0.95	-	-	-
FNJ	fnj -I phylip -O newick	n/a	1.0.1	-	-	-
RNJ	clearcut	n/a	1.0.9	-	-	-
DNJ	ccphylo tree -p	0 (0)	0.6.0	**1.82 TB**	**61:19:18.02**	**61:23:30**
DNJ^{t8}	ccphylo tree -pt 8	0 (0)	0.6.0	**1.82 TB**	98:28:50.51	69:04:56
HNJ	ccphylo tree -pm hnj	n/a	0.6.0	**1.82 TB**	74:00:12.66	74:13:09

Table S3; Computational requirements of NJ, RapidNJ, NINJA, FNJ, RNJ, DNJ and HNJ, on an AMD EPYC 7352 24-Core Processor with 2 TB memory. RF: Robinson-Foulds distance calculated with IQ-Tree and RAXML. n: number of taxa in dataset. lt: Time interval to load distance matrix (measured with ccphylo). t8: 8 threads. Bold indicates lowest time or lowest peak memory for each dataset, measured with GNU time. T: Terminated forcefully due to excessive computational requirements. -: Method terminated gracefully due to insufficient memory. Text in red indicates failed run or invalid output.
